# Sustained Post-Mating Response in *Drosophila* *melanogaster* Requires Multiple Seminal Fluid Proteins

**DOI:** 10.1371/journal.pgen.0030238

**Published:** 2007-12-14

**Authors:** K. Ravi Ram, Mariana F Wolfner

**Affiliations:** Department of Molecular Biology and Genetics, Biotechnology Building, Cornell University, Ithaca, New York, United States of America; North Carolina State University, United States of America

## Abstract

Successful reproduction is critical to pass genes to the next generation. Seminal proteins contribute to important reproductive processes that lead to fertilization in species ranging from insects to mammals. In *Drosophila,* the male's accessory gland is a source of seminal fluid proteins that affect the reproductive output of males and females by altering female post-mating behavior and physiology. Protein classes found in the seminal fluid of *Drosophila* are similar to those of other organisms, including mammals. By using RNA interference (RNAi) to knock down levels of individual accessory gland proteins (Acps), we investigated the role of 25 Acps in mediating three post-mating female responses: egg production, receptivity to remating and storage of sperm. We detected roles for five Acps in these post-mating responses. CG33943 is required for full stimulation of egg production on the first day after mating. Four other Acps (CG1652, CG1656, CG17575, and CG9997) appear to modulate the long-term response, which is the maintenance of post-mating behavior and physiological changes. The long-term post-mating response requires presence of sperm in storage and, until now, had been known to require only a single Acp. Here, we discovered several novel Acps together are required which together are required for sustained egg production, reduction in receptivity to remating of the mated female and for promotion of stored sperm release from the seminal receptacle. Our results also show that members of conserved protein classes found in seminal plasma from insects to mammals are essential for important reproductive processes.

## Author Summary

In sexually reproducing organisms, sperm enter the female in combination with seminal proteins that are critical for fertility. These proteins can activate sperm or enhance sperm storage within the female, and can improve the chance that sperm will fertilize eggs. Understanding the action of seminal proteins has potential utility in insect pest control and in the diagnosis of certain human infertilities. However, the precise function of very few seminal proteins is known. To address this, we knocked down the levels of 25 seminal proteins individually in male fruit flies, and tested the males' abilities to modulate egg production, sperm storage/release, or behavior of their mates. We found five seminal proteins that are necessary to elevate offspring production in mated females. Four of these proteins are needed for efficient release of sperm from storage to fertilize eggs, a function that had not been previously assigned to any seminal protein. All four are in biochemical classes that are conserved in seminal fluid from insects to humans, suggesting they may play similar sperm-related roles in other animals. In addition to assigning functions to particular seminal proteins, our results suggest that fruit flies can serve as a model with which to dissect the functions of conserved protein classes in seminal fluid.

## Introduction

Molecules in seminal fluid induce physiological changes in females, thereby affecting the reproductive capacity of both sexes ([1–6]; for reviews see [7–9]). In some animals, the absence or different levels of certain individual seminal proteins can lead to sterility (e.g., [10,11]). In others, including *Drosophila*, the absence of a particular seminal fluid protein can impair fertility and/or interfere with certain post-mating effects [12–15]. *Drosophila*, with its stereotyped mating behavior, excellent genetics, and characterized set of seminal fluid proteins, allows comprehensive tests for seminal fluid protein function.

In *Drosophila*, proteins synthesized and secreted by accessory glands of the male reproductive tract form part of the seminal fluid and are transferred to the female during mating [16–20]. These accessory gland proteins (Acps) induce striking physiological as well as behavioral changes in mated females (reviewed in [7–9]). These post-mating changes include increased egg laying (due to increased oogenesis [21] and increased ovulation [22]), the induction of genes encoding antimicrobial proteins [23–25], and decreased female receptivity to remating [3,26]. In addition, Acps are essential for causing morphological changes in the mated female reproductive tract [27] and for normal sperm storage and utilization in females [5,28], and they are hypothesized to play important roles in sperm competition [29–32]. Acps also form part of the mating plug [20,33]. Further, Acps have been implicated in reducing the life span [34,35] and influencing the feeding behavior [36] of the mated female.

The post-mating responses of increased egg production and reduction in receptivity to remating occur in two phases: a short-term and long-term response [3,5,37–39]. The short-term response occurs during the first 24 h after mating, with the induction of elevated egg production and reduction in receptivity largely dependent on Acps and not sperm. One Acp, ovulin (Acp26Aa), affects only short-term egg production by regulating ovulation for the first 24 h [13,22]. After 24 h, maintenance of post-mating physiological and behavioral changes requires the presence of stored sperm in the female (the long-term response; also called sperm effect [37,38]). One Acp, the sex peptide (SP, Acp70A), is known to be critical for the long-term mating response: females mated to SP null males fail to elevate egg production and remain highly receptive to remating [12,14]. A mechanism suggested for the SP-mediated long-term response is the binding of SP to sperm [40]. Subsequently, the C-terminal portion of the SP is released from the tail of sperm stored within the female. It is proposed that this released C-terminal SP is involved in eliciting the long-term response [25].

In Drosophila melanogaster, 112 predicted Acps (including SP and ovulin) have been identified so far by analyses of RNA, expressed sequence tags (EST), and recent proteomic and microarray analyses (see [9] for references and discussion). These molecules are predicted to belong to protein classes that include peptides and prohormones, lectins, lipases, proteases, protease inhibitors, cysteine rich secretory proteins (CRISPs), and defensin-like proteins [41]. Interestingly, similar protein classes are found in the seminal fluids of mammals [42–44], crickets [45,46], medflies [47], and honeybees [48]. Because *Drosophila* seminal fluid proteins are members of conserved families that are found in the seminal fluid of animals ranging from insects to mammals, *Drosophila* can potentially serve as a model system with which to dissect seminal fluid protein function genetically. However, biological functions of only a few Acps are known to date. These include three peptides and prohormones (ovulin, SP, and CG10433 [12–14,22,26,35,36,39,49–51]), three predicted or known Acp protease inhibitors (Acp62F [52]; CG8137, and CG9334, [51]), two predicted Acp proteases (CG11864, [53]; CG6168, [51]), and the glycoprotein Acp36DE [15,54,55]. To obtain a more comprehensive picture of the Acps that mediate post-mating changes and to understand how these changes are triggered mechanistically, it is essential to identify the functions of other Acps.

Here, we used RNA interference (RNAi) to systematically investigate the roles of Acps in inducing changes in egg laying, fertility, receptivity, and sperm storage. We focused on Acps within five predicted biochemical protein classes, members of which are known or suggested to be critical for several reproductive processes in *Drosophila* and/or mammals. We analyzed the functions of 25 Acp peptides/prohormones, lectins, CRISPs, proteases, and protease inhibitors; these comprise ∼50% of the stringently defined Acps [56]. We chose peptides/prohormones given the important roles of SP (peptide) and ovulin (prohormone-like Acp) in *Drosophila* reproduction (reviewed in [40,57]). Acps in the lectin, CRISP, protease, and protease inhibitor classes were included because of the conservation of these biochemical protein classes in the seminal fluids across various organisms, the important reproductive functions of some members of these classes in higher vertebrates including mammals (reviewed in [58]), and our previous finding of the importance of proteolysis regulators in *Drosophila* seminal protein processing [53].

We identified five Acps (a new member of the peptide class, member(s) of the lectin class, one CRISP, and one predicted protease) that affect egg production and fertility. Four of these Acps are also needed for persistence of the reduction of the mated female's receptivity to remating and for modulating the release of sperm from storage. Our findings on this latter group of four Acps indicate that multiple Acps are required for long-term post-mating responses to come into effect in mated females.

## Materials and Methods

### Flies

We used the *w^1118^* strain of D. melanogaster to generate transgenic lines and *tubulin*-GAL4/TM3, *Sb* flies [59] to generate knockdown (RNAi) or control males. Assays were done by crossing these knockdown or control males to females of the Canton-S strain of D. melanogaster. All flies were maintained on yeast-glucose medium at room temperature (22 ±1 °C) and a 12:12 light dark cycle.

### Generation of Transgenic Strains for Acp Knockdown Through RNAi

We initially focused our analysis on 26 Acps in five predicted protein functional classes (Table 1). Lines for the majority of Acps tested here were made following the method in Ravi Ram et al. [53]; generation of lines for the remaining Acps was reported in that paper, where those lines were tested for effects on ovulin processing. Briefly, using the Gateway system (Invitrogen), we moved each Acp into a modified form of the sympUAST vector [60], altered to accept Gateway inserts (sympUAST-GW, [53]). Transgenic fly lines carrying different sympUAST-Acp (UAS-Acp-UAS) constructs and subsequent experimental or knockdown males (*tubulin*-GAL4;UAS-Acp-UAS) as well as control males (*TM3, Sb;*UAS-Acp-UAS) were generated. Protein samples for western blotting were prepared by dissecting accessory glands from eight RNAi or control males and homogenizing them in the 40 μl 2× SDS sample buffer (as described in [20]); protein equivalent to the amount in one male was loaded into each gel lane. For all ten Acps for which sufficiently clean antibodies are available (Table 1), we used western blotting to detect the level of knockdown as in Ravi Ram et al. [20,53] For Acps for which no antibodies were available (Table 1), we used reverse transcription PCR (RT-PCR) to confirm knockdown at the transcript level, as in Chapman et al. [12] and Ravi Ram et al. [53].

**Table 1:**
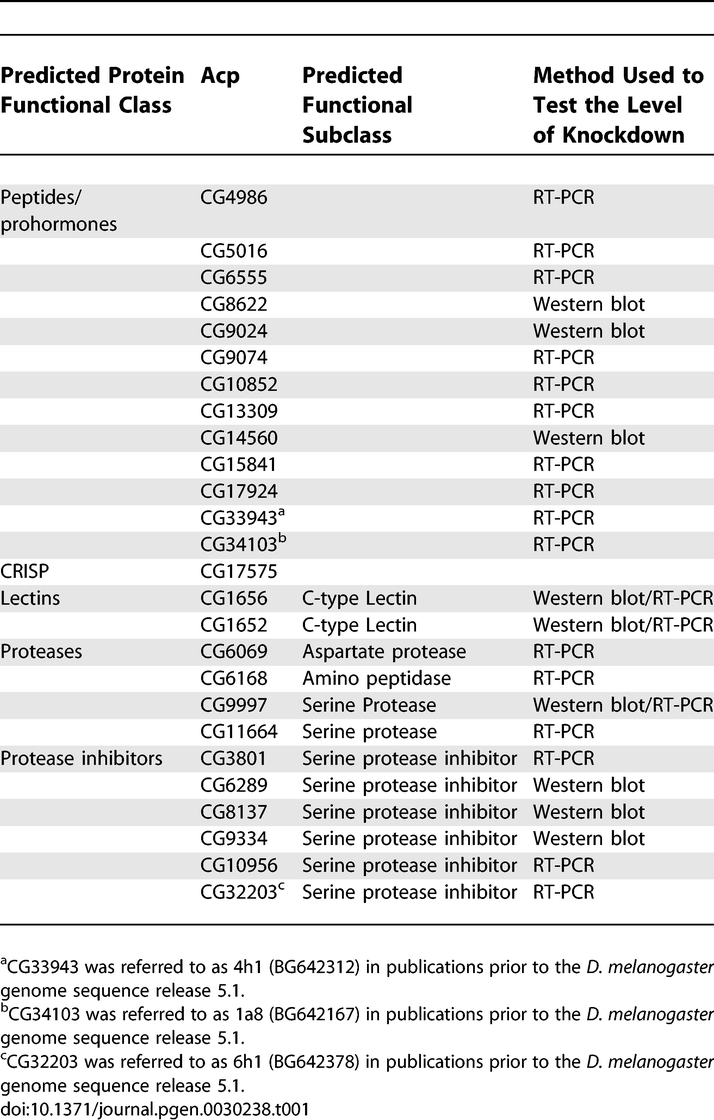
The Acps Knocked Down in the Present Study Fall Into Different Protein Classes

To check the specificity of gene targeting, the gene sequences used to generate dsRNA were subjected to the dsCheck software (http://dsCheck.RNAi.jp/; [61]). We also BLASTed the coding sequences of those Acps for which we detected phenotypes upon RNAi against the predicted genes data base (D. melanogaster genome release 5.1) using the BLASTn algorithm on the FlyBase BLAST server (http://flybase.net/blast/). To further confirm the specificity of the knockdowns and to analyze whether knockdown of any Acp affected the transfer of non-targeted seminal proteins to the mated female, we probed the proteins extracted from accessory glands of knockdown males, and from reproductive tracts of their mates, with other available Acp antibodies (for example, against CG14560, Acp26Aa, Acp36DE, Acp29AB, CG8137, CG6289, Acp53Ea, or CG9334 as in Ravi Ram et al. [20]). For 21 Acps, we observed no effects in males except for the knockdown of targeted Acp. For two pairs of gene duplicates (CG1652 and CG1656; CG8137 and CG9334), we observed no effects in males except for the knockdown of the targeted Acp and its duplicate (see below and [53]). However, for one Acp, CG9024, we observed that its knockdown affected the transfer of multiple Acps to the female (see Results/Discussion section for details). Therefore, of the 26 Acps with RNAi lines, we excluded CG9024 from further functional analysis and carried out the following functional assays using only the remaining 25 Acp knockdowns.

### Egg Laying, Fertility, and Hatchability Assays

Egg laying response (fecundity) of mates of knockdown or control males was quantified as described in Kalb et al. [3] and Herndon and Wolfner [13]. Fertility (number of progeny produced) and hatchability (by comparing the number of eggs laid to the number of progeny) assays were done as in Ravi Ram et al. [53]. The assays were calibrated using ovulin null and control males [13] and then used to assess RNAi males and their matched controls. For the initial screening, we carried out these assays twice each on one knockdown line per Acp construct for 25 Acps. Each time, 15–20 females mated to either control or experimental males were measured for egg laying and fertility. The differences in fecundity, fertility, or hatchability between the females mated to control or experimental males were statistically analyzed by Mann-Whitney U test using the JMP5.1 statistical program (SAS Institute, Cary, NC, USA). For Acp knockdown lines that showed a phenotype, we repeated the assay on a minimum of 2–3 independent lines per Acp construct to control for the insertion/line effect. In all cases, *TM3, Sb*;UAS-Acp-UAS male siblings of the experimental flies were used as controls. Experimental males of one line also acted as controls for other lines to rule out any possible effects due to the ubiquitous presence of GAL4 protein in the fly. A Bonferroni correction for multiple tests was performed for fecundity and fertility assays.

### Receptivity Assays

The receptivity response to remating for all 25 Acps tested was measured as in Kalb et al. [3] and Ravi Ram et al. [53] at 24 h ASM (after the start of mating) to test for short-term response. The assay was calibrated with SP null and control males [14] and subsequently assessed using RNAi males and their matched controls. For the Acp lines that showed a longer-term phenotype, receptivity to remating was also measured at 4 d ASM. A minimum of 15–20 females at 24 h ASM or at 4 d ASM were analyzed for control and experimental groups. Fisher's exact tests were used to determine significance and a Bonferroni correction was subsequently performed for multiple tests.

### Sperm Storage

Sperm counts were performed as in Neubaum and Wolfner [15] at 2 h ASM, 4 d ASM, and 10 d ASM. Each slide was counted twice to assess the counting precision which was >92.5%, and sample identity was coded to avoid bias. A minimum of 15–25 replicates were counted for each time point per treatment and data were analyzed using two tailed Student's t-test and subsequently a Bonferroni correction was applied for multiple tests.

## Results/Discussion

### Targeted Acps Are Knocked Down in RNAi Lines

**Figure 1:**
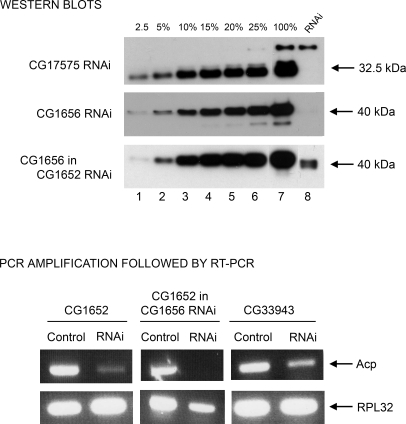
Example Western Blots and RT-PCR Showing the Levels of Acps in Knockdown Males (RNAi) Compared to Control Males In western blots, the extents of Acp knockdowns were quantified by running serial dilutions to the level of 2.5% (lane 1), 5% (lane 2), 10% (lane 3), 15% (lane 4) 20% (lane 5), 25% (lane 6) and 100% (lane 7) of accessory gland proteins from control males in parallel with accessory gland proteins equivalent to the amount in one knockdown male (RNAi). Two independent sets of samples were analyzed to confirm the level of knockdown. Additional multiple nonquantitative westerns were carried out on these lines to confirm their knockdown prior to each assay. Knockdowns were specific to the targeted Acp except for the related C-type lectins (CG1652 and CG1656). RNAi of CG1652 also knocks down CG1656, as shown. Similarly, knockdown of CG1656 knocks down CG1652; an example is shown using RT-PCR. For PCR amplification, cDNA prepared from RNA extracts of 20 control males or experimental males was used. RPL32 primers [30] were used as positive control for the quality/quantity of cDNA. The panel on the extreme right in the “RT-PCR” panel represents an example of knockdown specific to the targeted Acp (CG33943). We observed similar results for all other Acps tested either by western or RT-PCR (unpublished data).

We analyzed multiple independent RNAi lines for all 26 Acps. For all ten Acps for which sufficiently specific affinity purified antibodies are available to use on western blots, the levels of the targeted Acp detected on such blots was knocked down to ≤ 2.5% of control levels, although not to zero (here and [53]; see Figure 1 western blots panel for examples). For Acps with no available antibodies, we used RT-PCR and we observed no/little amplification of mRNAs from the RNAi targeted genes in knockdowns relative to control males (here and [53]; see Figure 1 PCR amplification panel for examples). Using the dsCheck program to check the specificity of gene targeting, we did not detect any potential off-targets for any targeted Acp except for the two pairs of gene duplicates mentioned in Materials and Methods (*CG1652* and *CG1656* is one pair; *CG8137* and *CG9334* is the other). In each pair, one member is the off-target of the other. These cases of off-targeting between gene duplicates are not surprising: *CG1652* and *CG1656* are about 75% identical [41,56], and *CG8137* and *CG9334* are about 85% identical [41,53,56] at the DNA level. Indeed, when we probed protein samples from accessory glands of CG1656 RNAi males with affinity-purified anti-CG1656, we observed that the levels of CG1656 were knocked down to ≤ 2.5% of the control males (Figure 1, western blots panel, see CG1656 RNAi) but in CG1652 RNAi males, the levels of CG1656 were knocked down only to ∼5%–10% relative to control males (Figure 1, western blots panel, see CG1656 in CG1652 RNAi). Using RT-PCR we observed that *CG1652* transcript levels were knocked down not only in CG1652 but also in CG1656 knockdown males (Figure 1, PCR amplification panel). In a separate study, we found that RNAi for the other pair of gene duplicates, *CG8137* and *CG9334*, also reciprocally knocked down each other [53]. We did not see inappropriate knockdown of any other Acps. For the Acps with common phenotypes, even in the BLAST search, with the exception of the members of gene duplicate pairs noted above, we did not detect any overlap in the genes pulled out of the BLASTn search (with sequence similarity of <30%), suggesting that the observed phenotypes are not due to any common off-target(s). Further tests on specificity of targeting using western blots showed that all knockdown males tested synthesized normal amounts of non-targeted Acps (see Figure S1 for examples). For 25 of the targeted Acps, all knockdown males transferred all tested Acps to their mates in normal amounts (See Figure S1 for examples).

However, although CG9024 (Acp26Ab) knockdown males made normal amounts of all tested Acps except CG9024 (Figure S1; see CG9024 under accessory gland panel), they transfer reduced amounts of all Acps tested (Figure S1; see CG9024 under mated female reproductive tract panel). We observed this with both independent lines of CG9024 knockdown males tested (Figure S1). Because we used *tubulin-GAL4* to drive the expression of UAS-9024-UAS, at present we do not know whether the reduced transfer is the consequence of CG9024 knockdown in accessory glands alone. Because of its reduced Acp transfer, we did not include CG9024 RNAi in assays of effects on females. Therefore, the results reported in this paper are for the remaining 25 Acp knockdowns.

### Multiple Acps Are Required for the Persistence of Post-Mating Changes in *Drosophila*

Mating triggers females to undergo both physiological and behavioral changes (examples reviewed in [7,9,58,62]). In D. melanogaster, these post-mating changes reflect the combined effects of sperm and proteins including Acps in the seminal fluid ([63,64]; for review see [57]). To determine the role of Acps in modulating the physiological and behavioral changes of the mated female, we analyzed the effects of individual knockdown of 25 Acps (see above) on egg laying, fertility, hatchability, receptivity, and storage of sperm in mated females.

**Figure 2:**
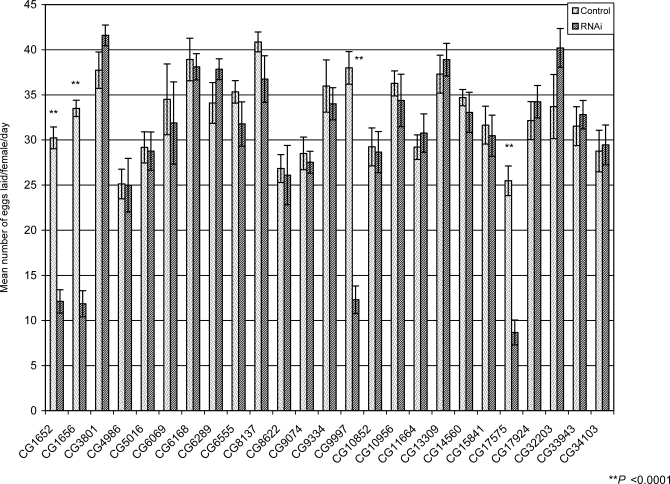
The Effect of Knockdown of Different Acps on Egg Laying by Mated Females Shown here is the number (mean ± SE) of eggs laid per day by the mates of knockdown males (RNAi) or control males over a period of 10 days. Egg laying response by the mates of most knockdown males was not significantly different from their control mates (*p* > 0.5). However, mates of CG1652/CG1656, CG17575, or CG9997 laid significantly fewer eggs compared to their controls (*p* < 0.0001). Number of females ranged from 15–45 depending on the Acp analyzed.

**Figure 3:**
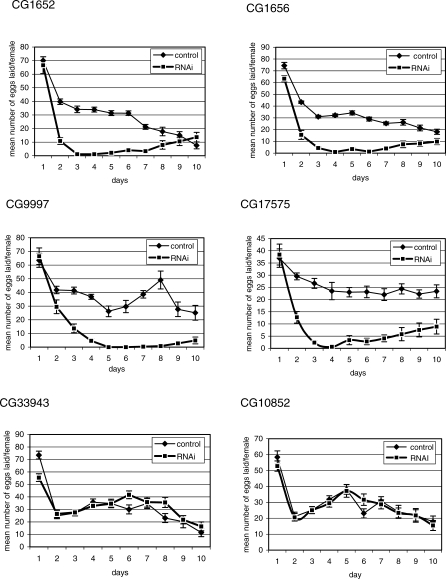
Mean Number (Mean ± SE) of Eggs Laid by the Mates of Knockdown Males (RNAi) or Control Males from Day 1 to Day 10 Egg laying by the mates of CG1652, CG1656, CG9997, or CG17575 knockdown males (RNAi) was not significantly different from their controls (*p* > 0.2) at 24 h ASM but from day 2 onwards, mates of CG1652, CG1656, CG17575, or CG9997 knockdown males laid significantly fewer eggs compared to their controls (*p* < 0.0001; *n* = 22–27). On the contrary, mates of CG33943 knockdown males laid significantly fewer eggs only at 24 h ASM (*p* < 0.0003; *n* = 30 for control and 27 for knockdown) compared to their controls; from day 2 onwards their levels were similar to the controls (overall *p* > 0.6). The other Acps did not differ from controls. One example is shown here (CG10852 panel; overall *p* > 0.8).

To identify new Acps necessary to stimulate these post-mating responses, we counted the number of eggs laid (fecundity), and the number of progeny produced (fertility), by females mated to Acp knockdown or control males. *Drosophila* females show post-mating responses of elevated levels of egg laying and reduced receptivity in two phases: a short-term response for one day is largely dependent on Acps, and a long-term response persisting for about 1–2 weeks requires both sperm and Acps [37,38]. Therefore, we analyzed fecundity/fertility data at 24 h ASM to identify Acps affecting the short-term response and total fecundity and fertility counts over a period of 10 days to identify Acps involved in the long-term response. Males depleted for CG33943, CG1652/CG1656 (gene duplicates), CG17575, or CG9997 did not stimulate full egg laying in their mates (Figures 2 and 3). Mates of the remaining 20 knockdown males did not significantly differ from controls in the overall number of eggs laid (*p* > 0.5; Bonferroni correction at the 5% level is 0.002; Figure 2).

#### CG33943 is required for full stimulation of egg laying on day 1 post mating.

Previous studies of one Acp, ovulin, showed that this Acp induces only a short-term response on egg laying: females mated to ovulin null males do not increase egg laying to the level of controls within the first 24 h ASM [13]. Ovulin is a prohormone-like molecule [19] that induces a short-term increase in ovulation [22]. To test whether any other Acps affect the short-term response, we analyzed egg laying by females mated to knockdown or control males. We found that CG33943 is necessary for increased egg laying levels at 24 h ASM. We observed that females mated to CG33943 knockdown males had reduced egg laying at 24 h ASM compared to their controls (*p* < 0.0003; Bonferroni correction at the 5% level is 0.002; Figure 3). However, from 2 d ASM on, the egg laying response of females mated to CG33943 knockdown males was similar to control levels (Figure 3). As a consequence of normal levels of egg laying on days 2–10 ASM, the total fecundity of females mated to CG33943 knockdown males over a period of 10 days did not differ significantly from controls (*p* > 0.6; Bonferroni correction at the 5% level is 0.002; Figure 2). The percent hatchability of the eggs laid by the mates of knockdown males is similar to their controls (unpublished data). Therefore, the fertility of females mated to CG33943 knockdown males was affected only at 24 h ASM as a consequence of reduced egg laying, and fertility over a period of 10 days did not significantly differ from controls (*p* > 0.6; Bonferroni correction at the 5% level is 0.002; see Figure S2). These results are analogous to what was seen in similar experiments involving ovulin [13].

CG33943 is a novel peptide with no predictable domains in its sequence [41]. It is possible that CG33943 may act in concert with ovulin to induce a short-term response or may function independently of ovulin in the egg laying process. Further studies are required to identify the mechanism by which CG33943 induces a short-term response on egg production.

#### Two Acps and an additional pair of related Acps are required for the persistence of post-mating responses.

Females mated to CG1652/CG1656, CG17575, or CG9997 knockdown males had levels of egg laying similar to controls at 24 h ASM. That egg laying is elevated in females mated to CG1652/CG1656, CG17575, or CG9997 knockdown males in a way similar to their controls at 24 h ASM suggest that these Acps are not essential for the short-term responses, although these results could also be attributed to the fact that these flies are single Acp knockdowns that contain residual levels of targeted protein (as the levels are only knocked down to <2.5%) or to the presence of other redundant Acps, such as SP or ovulin.

However, from days 2–10 ASM, the number of eggs laid by the mates of CG1652/CG1656, CG17575, or CG9997 knockdown males was reduced significantly compared to controls (*p* < 0.0001; Bonferroni correction at the 5% level is 0.002; Figure 3). Consistent with their reduced egg laying, we found that females mated to CG1652/CG1656, CG17575, or CG9997 knockdown males had significantly lower fertility compared to controls (*p* < 0.0001; Bonferroni correction at the 5% level is 0.002; See Figure S2). Fertility of females mated to the remaining 21 knockdown males did not significantly differ from controls (*p* > 0.1; Bonferroni correction at the 5% level is 0.002; See Figure S2). CG1652 and CG1656 are gene duplicates [41] and they reciprocally knock down (see above). Therefore, it is possible that the observed reduction in egg laying and fertility may be caused by reduction of either CG1652 or CG1656 or both.

To determine whether the lack of any Acp affected hatchability of eggs and to test whether egg hatchability contributes to reduced fertility in mates of CG1652/CG1656, CG17575, or CG9997 knockdown males, we determined the percentage egg hatchability by comparing the number of eggs laid to the number of progeny. Knockdown of any of these Acps, or any of the other 21 Acps, did not affect hatchability (unpublished data) as the eggs laid by females mated to any knockdown males had hatchability levels comparable to controls. Therefore, our results of the reduced fertility of mates of CG1652/CG1656, CG17575, or CG9997 knockdown males appears to be a consequence of reduced egg production.

Previous studies showed that the long-term post-mating response also includes continued reduction of the receptivity of the mated female to remating [3,26]. To test whether CG1652/CG1656, CG17575, or CG9997 affected only egg laying or also other aspects of post-mating response, we tested the receptivity levels of females mated to these knockdown males and other Acp knockdown males at 24 h ASM. None of the 25 Acps (including CG33943, CG1652/CG1656, CG17575, and CG9997) appeared to modulate the receptivity of the mated female at 24 h ASM (Table 2): females mated to either control or knockdown males showed equally low receptivity to remating (*p* > 0.2; Bonferroni correction at the 5% level is 0.002). Since we observed that the long-term fecundity is affected in females mated to CG1652/CG1656, CG17575, or CG9997 knockdown males, we tested if long-term receptivity is affected in these females by analyzing their receptivity to remating at 4 d ASM. At 4 d ASM, mates of CG1652/CG1656, CG17575, or CG9997 knockdown males were significantly more receptive to remating (Table 3) than mates of control males (*p* < 0.005; Bonferroni correction at the 5% level is 0.01). In contrast, females mated to CG33943 knockdown males included as a control show normal response and were as receptive as their controls (*p* > 0.3; Bonferroni correction at the 5% level is 0.01). These results suggest that CG1652/CG1656, CG17575, and CG9997 are required for the sustained reduction of the receptivity to remating of the mated female. That these Acps affect both egg laying and receptivity, and do so for a sustained period, indicates that these Acps are necessary in the long-term persistence of post-mating changes of mated females.

**Table 2:**
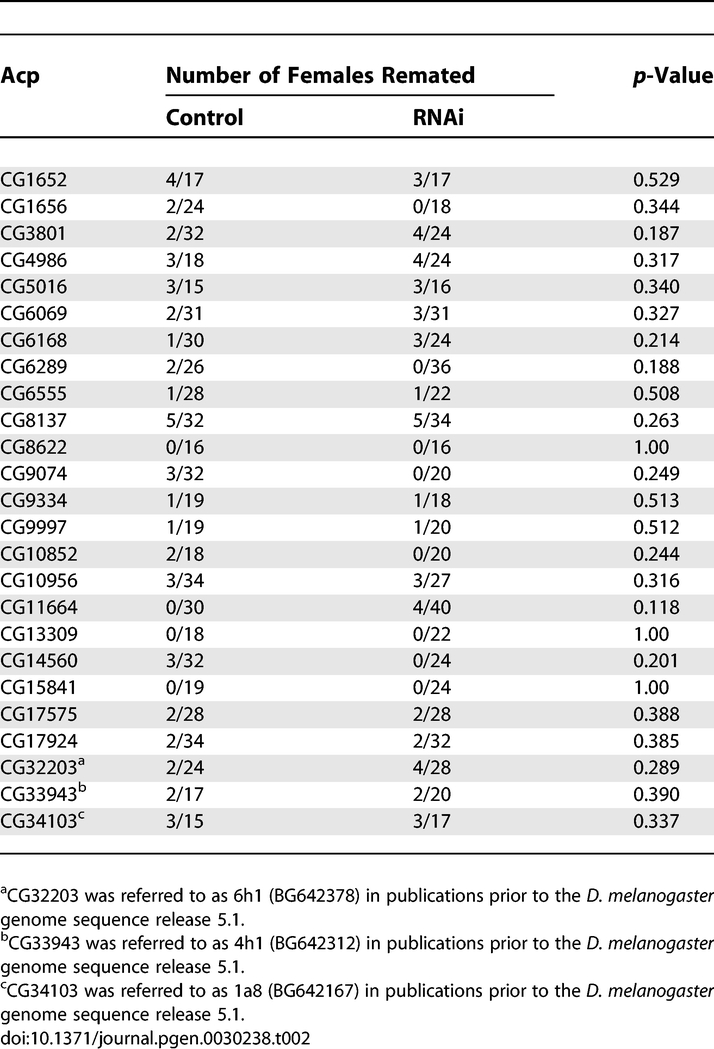
Remating Efficiency of Females Once Mated to Knockdown (RNAi) Males or Control Males at 24 h after First Mating (24 h ASM)

**Table 3:**
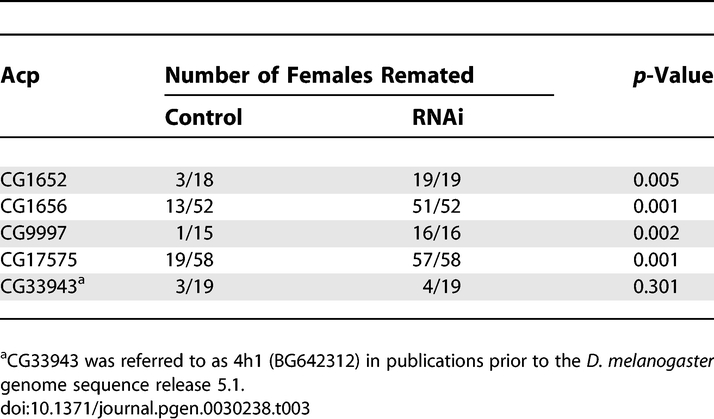
Remating Efficiency of Females Once Mated to Knockdown (RNAi) Males or Control Males at 4 d after First Mating (4 d ASM)

### CG1652/CG1656, CG17575, and CG9997 Affect Release of Sperm from Storage

Sperm in storage is a major factor in the long-term persistence of post-mating responses [37,38]. Since mates of CG1652/CG1656, CG17575, or CG9997 knockdown males were defective in the long-term post-mating response, we tested whether these knockdowns affected sperm storage.

D. melanogaster females store sperm in the single seminal receptacle (containing about 65%–80% of the stored sperm) and the paired spermathecae [15,28,65,66]. Acps are required for normal sperm storage and utilization in the mated female [5,15,28].

**Figure 4:**
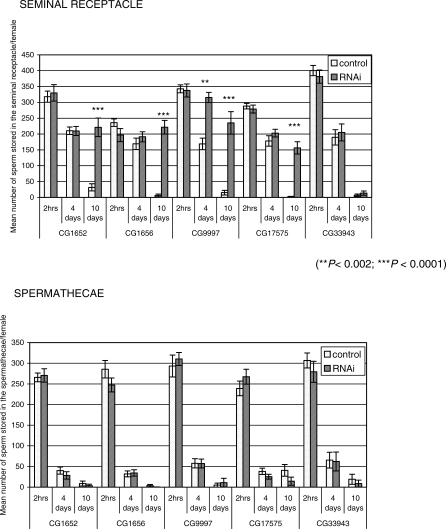
Number of Sperm Stored (Mean ± SE) in Seminal Receptacle or Spermathecae of Mates of CG1652, CG1656, CG9997, or CG17575 Knockdown Males (RNAi) Compared to their Controls at 2 h ASM, 4 d ASM, and 10 d ASM. (A) We did not detect a significant difference in the number of sperm stored by mates of CG1652, CG1656, or CG17575 knockdown males compared to their controls in the seminal receptacles at 2 h ASM (*p* > 0.25) or 4 d ASM (*p* > 0.2) but we observed significant difference at 10 d ASM (*p* < 0.0001). Mates of CG9997 knockdown males had sperm storage levels similar to their controls at 2 h ASM (*p* > 0.2) but at 4 d and 10 d ASM, the number of sperm stored in the seminal receptacle of mates of CG9997 knockdown males was significantly higher than their controls (*p* < 0.0001). (B) We did not detect significant difference in the number of sperm stored by mates of CG1652, CG1656, CG9997, or CG17575 knockdown males compared to their controls in the spermathecae at 2 h ASM (*p* > 0.2) or 4 d ASM (*p* > 0.2) or 10 d ASM (*p* > 0.5). Number of females ranged from 15–35 per treatment depending on the Acp analyzed or the storage organ counted.

We counted the number of sperm in the sperm storage organs of females mated to CG1652/CG1656, CG17575, or CG9997 knockdown males along with their controls at several times post-mating. Mates of any of these Acp knockdown or control males did not differ significantly in the number of sperm in storage at 2 h ASM (*p* > 0.2; Bonferroni correction at the 5% level is 0.01; Figure 4, seminal receptacle panel). At 4 d ASM, there was no significant difference in the number of sperm stored in the seminal receptacle of females mated to CG1652/CG1656, CG17575, or CG33943 knockdown males compared to their controls (*p* > 0.2; Bonferroni correction at the 5% level is 0.01; Figure 4, seminal receptacle panel). However, at 4 d ASM, females mated to CG9997 knockdown males had significantly more sperm stored in the seminal receptacle when compared to their controls (*p* < 0.002; Bonferroni correction at the 5% level is 0.01; Figure 4, seminal receptacle panel). Interestingly by 10 d ASM, females mated to CG1652/CG1656 or CG17575 knockdown males, as well as mates of CG9997 knockdown males, had significantly more sperm stored in their seminal receptacle compared to their controls (*p* < 0.0001; Bonferroni correction at the 5% level is 0.01; Figure 4 seminal receptacle panel). These results suggest that sperm from CG1652/CG1656, CG17575, or CG9997 knockdown males can get into storage as efficiently as sperm from their control males but that sperm from knockdown males are not released from the seminal receptacle as efficiently as sperm of control males. These results also suggest that like their effect on long-term egg production and receptivity, CG1652/CG1656, CG17575, or CG9997 affect the long-term release of sperm from storage. We observed no significant difference in the number of sperm stored in the spermathecae of females mated to any of these knockdowns or control males (*p* > 0.2; Bonferroni correction at the 5% level is 0.01; Figure 4, spermathecae panel) at all three time points. The egg hatchability of mates of CG1652/CG1656, CG17575, or CG9997 knockdown males was unaffected (above and unpublished data), suggesting that the sperm from these RNAi males in storage are of normal viability and have normal capacity to fertilize eggs.

Despite the presence of viable sperm in females mated to males knocked down for CG1652/CG1656, CG17575, or CG9997, these females were deficient in sustaining long-term egg production and were receptive to remating. This is similar to findings previously reported for another Acp, SP [12,14]. Chapman et al. [12] and Liu and Kubli [14] proposed that the sperm effect is in fact an effect of SP, but one that is manifest only in the presence of sperm. In the present study, though knockdown males of each of the four Acps (CG1652/CG1656, CG9997, or CG17575) transferred normal levels of SP (unpublished data) to the mated females, those females still failed to exhibit the long-term post-mating responses. These results suggest that manifestation of long-term post-mating responses requires multiple Acps (CG17575, CG1652/CG1656, and CG9997 as well as SP [39]) and that this occurs only in the presence of sperm. The four Acps identified here may either act in concert with each other and/or SP or may act in independent pathways to promote long-term mating response.

Storage and management of sperm within females is a multistep process (reviewed in [67]) involving progression of transferred sperm through the female reproductive tract towards the storage organs, entry of sperm into the storage organs, maintenance of viable sperm in storage, and release of sperm from storage for fertilization. One Acp, Acp36DE, is essential for the entry of sperm into storage [15,54]. However, the role of Acps in the release or utilization of stored sperm is poorly understood. The present study has provided candidate Acps for future studies to understand the role of Acps in sperm management. Females mated to CG1652/CG1656, CG17575, or CG9997 knockdown males had significantly more sperm in the seminal receptacle at later time points, suggesting a requirement of these Acp(s) for the release of sperm from storage. However, the observation that the release of sperm only from the seminal receptacle, but not from the spermathecae, is affected in mates of these RNAi males is intriguing. At present not much is known about the pattern of utilization of sperm from these storage organs in *Drosophila* except that spermathecae have been suggested to serve as long-term storage organ [65]. However, it is interesting to note that CG1652/CG1656, CG17575, and CG9997 affect both sperm release and egg laying. It is not clear whether and how these latter two phenotypes are linked. Release of sperm from storage is independent of presence of eggs in the long-term [68], although presence of eggs affects some transitions in the sperm storage process. It is not known whether egg laying rate is affected by the rate of sperm release. Consideration of tissue targets of these Acps may be informative. Targets of CG1652, CG1656, CG9997, and CG17575 have been identified ([20], Ravi Ram and Wolfner, unpublished data). All four are detected in the uterus immediately after mating where they can presumably interact with each other, with other Acps, and with sperm. CG1652, CG1656, and CG9997 are subsequently detected in sperm storage organs, consistent with a role in affecting these tissues and/or the sperm stored within them. Further studies of the interactions between these Acps (and SP), if any, and of their specific localization may provide an insight into mechanisms of controlling sperm release from storage, and its interaction with egg laying.

### Members of Conserved Protein Classes in the Seminal Fluids Are Critical for Reproduction Across Organisms

The Acps for which we have identified roles in sperm release fall into three different protein classes. CG1652 and CG1656 are predicted C-type lectins, CG17575 is a predicted CRISP, and CG9997 is a predicted serine protease [41]. These protein classes are found in seminal fluids across wide range of taxa (*Drosophila*, [9,41]; mammals [42–44]; crickets, [45,46]; medflies, [47]; honeybees, [48]; see [9,58] for reviews). In vertebrates, including mammals, lectin-like spermadhesins (reviewed in [69–71]), CRISPS [72–74], and proteases ([75,76] and reviewed in [77]) in the seminal fluid are suggested to play important roles in reproductive processes such as sperm function and mediating gamete fusion. In the present study, we showed that member(s) of these families in *Drosophila* seminal fluids are important for sustained post-mating responses. A separate study demonstrated that a predicted astacin-like Acp protease plays an important role in proteolysis of seminal fluid molecules [53]. Therefore, members of the conserved protein classes in the seminal fluid are critical for successful reproduction in *Drosophila* as well as in vertebrates. It will be interesting in future studies to determine if the similar protein classes are involved in analogous mechanisms across different organisms.

To conclude, we have found that *Drosophila* seminal protein CG33943 plays an essential role in short-term induction of egg laying. We also found that seminal proteins CG1652/CG1656, CG9997, and CG17575 are essential for sustained egg laying, reduced receptivity of the mated female, and for the release of sperm from storage, indicating that the maintenance of female post-mating responses for a long term requires multiple Acps contributed by the male. The observations from the present study, along with those of previous studies, show that some Acps act only to mediate short-term responses while others are required for long-term post-mating responses. Lack of any significant phenotype for the remaining 20 Acp knockdowns does not mean that they lack reproductive function, but instead could be due to (a) their functions in processes that we did not assay, such as regulation of sperm competition [32], cost of mating (longevity, [34,35]), female post-mating feeding behavior [36], or in inducing female immune response [25,51], (b) remaining residual levels of the targeted Acp, since RNAi knocks down Acp levels but does not completely remove them; residual levels might be sufficient to mediate post-mating changes or might have caused changes smaller than our level of detection, (c) redundancy in Acp function. Redundancy has already been observed for some Acps' function: although both ovulin [13] and SP [12,14] are essential to increase egg laying post-mating, removal of either one does not completely abolish the increase in egg laying. Further, there is a redundancy in tissue targeting of Acps: more than one Acp targets to any given tissue in the mated female reproductive tract [20]. Further studies involving the knockdown of these different Acps in multiple combinations may help in the identification of their functions. Finally, our results suggest that members of conserved protein classes in the seminal fluid are critical for optimal reproductive output and therefore that *Drosophila*'s suite of seminal proteins can serve as a potential model for understanding the roles of these conserved protein classes in the seminal fluid.

## Supporting Information

Figure S1 Analysis of Effect of Acp Knockdowns in the Synthesis and Transfer of Acps to Females During Mating To test whether knockdown of any of the Acps affects the synthesis and the transfer of Acps to female, we probed protein samples from male accessory glands of the knockdown males (RNAi) and control males, and from female reproductive tracts of mates of each type of male, respectively. Subsequent western blotting showed that the level of non-targeted Acps is normal in all the knockdown males (Accessory gland panel). Further, mates of all different knockdown males (RNAi) received Acps to the level comparable to control males except for mates of CG9024 knockdown males (lanes RNAi for two different lines (AM and GM) of CG9024; mated female panel). Protein amounts equivalent to one accessory gland or two mated female reproductive tracts were loaded in each lane.
(480 KB TIF)

Figure S2 The Effect of Acp Knockdown on the Offspring Number (Fertility) of Mated Females Shown here are the mean progeny counts (mean ± SE) per day of the mates of knockdown males (RNAi) or control males over a period of 10 days. Fertility of the mates of most knockdown males was similar to their control mates (*p* > 0.1). Only the mates of CG1652/CG1656, CG17575, or CG9997 had significantly fewer progeny compared to their controls (*p* < 0.0001; the number of females ranged from 15–45 per treatment depending on the Acp analyzed).
(40 KB PPT)

## Abbreviations

Acp: accessory gland protein
ASM: after start of mating
CRISP: cysteine rich secretory protein
RNAi: RNA interference
RT-PCR: reverse transcription PCR
SE: standard error
